# Taylor on Poisons

**Published:** 1848

**Authors:** 


### ARTICLE III.

*On Poisons, in relation to Medical Jurisprudence and Medicine.* By ALFRED S. TAYLOR, F.R.S., Lecturer on Medical Jurisprudence in Gray's Hospital, and author of Medical Jurisprudence. Edited, with notes and additions, by R. EAGLESFIELD GRIFFITH, M.D., &c. pp. 687 8vo. Philadelphia: Lea & Blanchard. 1848. (From the Publishers.)

The excellent volume before us is an American edition of an English work—one of the best in the languaage—on toxicology, in its relations to law and medicine. It contains all that is known of poisons up to the present day—their properties and appearance; symptoms produced by them in varied doses; chemical tests—the latest and most approved; value of medical evidence in trials for poisoning; rules of conduct for medical men in ante-mortem and post-mortem examinations, &c.—all amply illustrated by citations of cases.

The subject of general poisoning is first treated of very fully; the subject is then classified, and each class separately noticed, first generally, afterwards each individual of the class in detail. It offers to the medical jurist, in itself, almost all the information that he could require, or could obtain, in relation to poisons, from a library of periodicals, law reports, and works on medical jurisprudence, chemistry, or materia medica. The American editor has enriched the original work by additions from, and reference to, many other works and periodicals, principally American, to which, probably, the author had not access. The volume is thus rendered complete in all respects, and, to the American practitioner and jurist, essentially increased in value. The publishers have done justice in their department, to the value of the work, by expending upon it the best materials and mechanical execution. Having experienced its value in a recent case of trial for poisoning, in which many intricate points of legal, medical, and chemical evidence were involved, in which we were cited as a medical witness, we feel that we cannot too highly recommend it to all who wish to be accomplished toxicologists, or have any desire to do themselves and the profession credit, and the public justice, when concerned as physician, witness, or counsel, in cases of poisoning. J. V. Z. B.

